# Decoding subtle forearm flexions using fractal features of surface electromyogram from single and multiple sensors

**DOI:** 10.1186/1743-0003-7-53

**Published:** 2010-10-21

**Authors:** Sridhar Poosapadi Arjunan, Dinesh Kant Kumar

**Affiliations:** 1 Bio-signals Lab, School of Electrical and Computer Engineering, RMIT University, GPO Box 2476, Melbourne, Victoria 3001, Australia

## Abstract

**Background:**

Identifying finger and wrist flexion based actions using a single channel surface electromyogram (sEMG) can lead to a number of applications such as sEMG based controllers for near elbow amputees, human computer interface (HCI) devices for elderly and for defence personnel. These are currently infeasible because classification of sEMG is unreliable when the level of muscle contraction is low and there are multiple active muscles. The presence of noise and cross-talk from closely located and simultaneously active muscles is exaggerated when muscles are weakly active such as during sustained wrist and finger flexion. This paper reports the use of fractal properties of sEMG to reliably identify individual wrist and finger flexion, overcoming the earlier shortcomings.

**Methods:**

SEMG signal was recorded when the participant maintained pre-specified wrist and finger flexion movements for a period of time. Various established sEMG signal parameters such as root mean square (RMS), Mean absolute value (MAV), Variance (VAR) and Waveform length (WL) and the proposed fractal features: fractal dimension (FD) and maximum fractal length (MFL) were computed. Multi-variant analysis of variance (MANOVA) was conducted to determine the *p *value, indicative of the significance of the relationships between each of these parameters with the wrist and finger flexions. Classification accuracy was also computed using the trained artificial neural network (ANN) classifier to decode the desired subtle movements.

**Results:**

The results indicate that the *p *value for the proposed feature set consisting of FD and MFL of single channel sEMG was 0.0001 while that of various combinations of the five established features ranged between 0.009 - 0.0172. From the accuracy of classification by the ANN, the average accuracy in identifying the wrist and finger flexions using the proposed feature set of *single *channel sEMG was 90%, while the average accuracy when using a combination of other features ranged between 58% and 73%.

**Conclusions:**

The results show that the MFL and FD of a single channel sEMG recorded from the forearm can be used to accurately identify a set of finger and wrist flexions even when the muscle activity is very weak. A comparison with other features demonstrates that this feature set offers a dramatic improvement in the accuracy of identification of the wrist and finger movements. It is proposed that such a system could be used to control a prosthetic hand or for a human computer interface.

## Background

Controlling devices such as prosthetic or robotic hand requires automated identification of the command. To provide the user with a natural feeling and benefit of the dexterity of the hand, the (intended) finger and hand movements of the user have to be identified. One method to determine movement and posture is by estimating the strength of contraction of associated muscles based on the electrical activity of the muscles. The benefit of this over other sensing techniques is that it is suitable for people who have suffered amputation of their hands and the control can be based on the user's intention. A recent survey on myoelectric prosthesis by Oskoei and Hu [1] has reported that the applications of sEMG based control are widespread such as multifunction prosthesis, wheelchairs, grasping control, virtual keyboards, and gesture-based interfaces.

Surface electromyography (sEMG) [2,3] is a non-invasive, easy to record electrical activity of the skeletal muscles recorded from the skin surface. Classification of sEMG to identify the hand movement and gesture is a desired option [4-7]. Identified hand and finger actions can be used to command robotic and prosthetic hand which will allow the users to benefit from high degrees of freedom offered by recently developed devices such as Shadow Robot hand (Shadow Company, London). However identification of the gestures and movements is not simple when there are a number of simultaneously active muscles and the muscle activity is weak such as during finger and wrist flexion and extension. To overcome this, often sEMG based commands are treated as binary and the user need to give a series of commands for functionality [8]. While able to overcome the issue of noise, the system is not natural and very limiting. As a result, modern prosthetic hands such as I-Limb (Touchbionics, Scotland) and Kinetic Human-type (KH) Hand S1 (Dainichi, Japan), while having the provision of controlling individual fingers, are able to provide only limited control to the user. The user can perform hand grasp actions where all the fingers move together and is unable to benefit from the control of individual fingers. If the finger and wrist actions of the user could be reliably identified from sEMG, such systems could be controlled by sEMG of the user and these devices would become very useful and more readily acceptable.

The fundamental principle underlying the identification of actions and gestures using sEMG is by measuring the strength of contraction of the associated muscles. This may be done in time or frequency domain or a combination. Various analogous measures such as root mean square (RMS), integral of the signal, auto-regression, signal length and wavelet coefficients have been used to identify the movement and/or posture [5,9-13]. The classification of these features has been achieved using a range of parametric and non-parametric techniques, such as Bayesian statistical classifiers, artificial neural networks (ANN) [10,13,14], support vector machine (SVM) [9] and predictive approach [12].

To identify actions that are a result of multiple simultaneously active muscles require an estimation of the relative strength of contraction of the different muscles. Researchers have used artificial intelligence and genetic algorithms [5,9,10,13,14] or an array of electrodes to estimate the relative strength of contraction based on the spatial distribution [7,10,15]. While these studies have successfully used multiple channels for identifying actions such as subtle finger and wrist movements, these have limited applications because of the need for precise location of the electrodes and most of these systems need to be calibrated for each session [16].

Recent work [9] has compared number of features of sEMG and classified these using SVM to determine the most effective set of features for identifying the hand actions for controlling the prosthetic devices. While it is a very useful evaluation of the different features, however it has limited applications because the system requires training for each session and only suitable for user selected actions. A comparison of the different features that have been proposed in recent literature [5,9] shows that these features are sensitive to the experimental conditions. There is a need for a simple and reliable system that does not require large number of electrodes is easy to use and does not require the system to be trained for each session.

One general limitation of the established and widely reported features of sEMG is that these are unreliable at low levels of contraction due to low signal-to-noise ratio [17]. At low level of contraction the relationship between sEMG and the force of contraction is not linear [17] and the signal to noise ratio for sEMG is very poor. Due to this reason, it is difficult to automatically segment the muscle activity from the background activity [3,18]. While statistical based segmentation techniques are suitable when the muscle activity is large, manual selection of the muscle activation period is required when muscle activity is small.

When the muscle activity is low, the density of motor unit action potentials (MUAP) can be used to determine the strength of muscle contraction [19,20]. The fundamental principle of determining MUAP density reported in literature [11,14,20,22,23] is based on shape matching. Strategies used include template matching [11], use of neural networks [10,13-15,18] and wavelet decomposition [12]. While such systems are suitable to be trained for any shape of MUAP but these are sensitive to changes in these shapes after training. Due to the differences in the conduction pathways of MUAPs originating from different muscles, there would be a variation in the shape of MUAP in the recordings at the surface. This makes shape based MUAP identifying techniques unsuitable when there are multiple active muscles.

Another proposed measure of strength of muscle activity is the fractal dimension (FD) of sEMG [4,24-28]. Fractals refer to properties of objects or signal patterns that exhibit self-similarity over a range of magnification/scales and with the relationship that is fractional. FD is a measure of this relationship and is estimated as the change in length of the curve with the change in the measurement scale. FD is a measure of the source properties and is a measure of its complexity, spatial extent or its space filling capacity and is related to shape and dimensionality of the process [29-31]. Gitter and Czerniecki [26] have reported that FD of the EMG signal correlates with muscle force. Gupta et al [27] have also reported that FD could be used to characterize the EMG signal.

**Figure 1:**
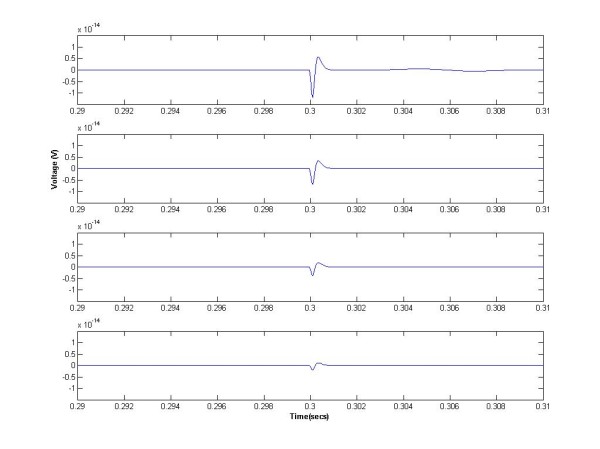
**Estimated shape of MUAPs at the surface when they originate from different distances from the electrodes**. The top trace shows the superficial muscle (10 mm from surface) and the last trace shows the deeper muscle (30 mm from surface). Simulated based on [10].

Based on the fundamental model of sEMG [10], MUAPs originating from superficial muscles have higher frequency and magnitude at the surface compared to the MUAPs originating from deeper muscles. Figure 1 shows the estimated shape of MUAPs at the surface when they originate from different distances (10 mm and 30 mm) from the electrodes [10]. Preliminary experiments [33] have shown that the FD of sEMG resulting from deeper muscles is significantly less than from superficial muscles, and signal from very distant muscles do not exhibit fractal properties. While high level of contraction or muscle stretch could also have an impact on FD [27], there would not be any significant variations of FD of a muscle with small changes of muscle contraction.

**Figure 2:**
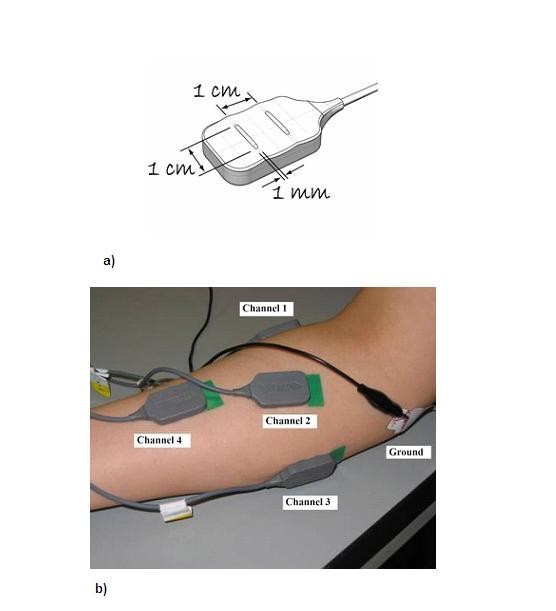
**(a) Bipolar electrode design (Source: DELSYS) and (b) placement of four bipolar electrodes on the surface of the forearm**.

A new feature, the maximum fractal length (MFL) of the signal, as a measure of the muscle activation has been proposed. This is the intercept of the fractal relationship with the length of the curve at lower scale (Figure 2). This is similar to the wavelength but because it is on the logarithmic scale, it is less sensitive to background noise and is a good indicator of the density of MUAPs irrespective of the shape [32]. Preliminary experiments [32,33] indicate that it is an easy to identify change in MFL in response to muscle contraction, and thus can be used to segment the muscle activity from the background activity.

Authors have identified the maximum fractal length (MFL) as a measure of the strength of contraction [32,33]. While similar to the wave-length [9], MFL incorporates the logarithmic scale, making it less sensitive to noise. Based on the above, it is hypothesized that the feature set consisting of FD and MFL of single channel sEMG are indicators of relative strength of contraction of the associated muscles and thus can accurately identify actions with multiple muscles and low-level contractions such as finger and wrist flexions. This paper reports the testing of this hypothesis. This feature set has also been compared with number of other features reported in literature [9].

## Methods

The aim of this work was to develop a sEMG based technique that can accurately identify the basic hand and wrist gestures which may be used by people in special circumstances to communicate or give commands. This requires determining suitable features of sEMG that can be used to identify the actions even when the muscle activity is low. It is also important to differentiate between activities of different muscles.

SEMG is the electrical recording resulting from the interferential summation of MUAPs at the surface. All MUAPs are similar at the source [11,14,20-23,34] suggesting the self-similarity in the signal. Differences arise in the shape and spectral content due to attenuation of the signal and the spatial filtering characteristics of the tissues through which the signal travels. Work reported by Gupta et al [27] and Gitter and Czerniecki [26] have demonstrated that sEMG signal has fractal properties. Based on this self-similarity, it is hypothesised that the fractal dimension of sEMG would indicate the property (such as size and complexity) of the active muscle while the MFL of the signal would indicate the MUAP density [32,33]. A combination of these can be used to identify different hand movements for controlling a prosthetic hand or a powered device.

### Subjects

Five subjects (four male and one female) volunteered to participate in this study. Mean age was 26.6 (*σ *= 2.05) years; mean weight 70.6 (*σ *= 6.56) kg; and mean height was 170.6 (*σ *= 7.42) cm. The participants' inclusion criterion was; (i) healthy with no history of myo or neuro-pathology, and (ii) no evident abnormal motion restriction. All participants in this study were right-handed. Experiments were conducted after receiving approval from University Ethics Committee for Human Experiments. Each participant was given an oral and written summary of the experimental protocol and the purpose of the study and then was required to sign a consent form prior to participation.

### EMG Recording Procedures

Four bipolar electrodes were placed on the following forearm muscles [35] as shown in Figure 2 and in accordance with standard procedures [11,36,37] to record surface electromyogram (sEMG):

Channel 1: Brachioradialis

Channel 2: Flexor Carpi Radialis (FCR)

Channel 3: Flexor Carpi Ulnaris (FCU)

Channel 4: Flexor digitorum superficialis (FDS)

DELSYS (Boston, MA, USA), a proprietary sEMG acquisition system, was used for recording sEMG. This system has bipolar differential electrodes units with each unit having two parallel bars with fixed inter-electrode distance of 10 mm (Figure 2) and a preamplifier with gain of 1000, notch filter of 50 Hz and associated harmonics and with 8^th ^order butterworth band pass filter from 20 to 450 Hz. The sampling rate of the system is 1024 samples/second for each channel. Prior to placing the electrodes, the skin of the participant was prepared by shaving (if required) and exfoliation to remove dead skin. Skin was cleaned with 70% v/v alcohol swab to remove any oil or dust from the skin surface. The skin impedance during the recording was measured and in all cases was less than 6 KΩ. Standard electrode placement procedures were followed [2,36,37].

### Experimental Protocol

At the start of the experiment, the participants were given a demonstration towards maintaining finger and wrist flexion. Prior to the recording, the participants were encouraged to familiarize themselves with the experimental protocol and with the equipment. SEMG was recorded from the four electrodes when the participant maintained the specific wrist and finger flexions: *M1 - All fingers and wrist flexion, M2 - Index and Middle finger flexion, M3 - Wrist flexion towards little finger, M4 - Little and ring finger flexion. *The flexions were performed without any resistance and as were convenient to and easily reproducible by the participant.

The recordings from four channels were used to compare with other similar studies, however for the single channel analysis, only channel 2, located closest to the elbow (Figure 2) was considered. The command for the action was displayed on the screen as well as given verbally. The order of the flexions was arbitrary and each flexion was maintained for about 7-8 seconds to obtain sEMG recordings during isometric contraction. Each flexion was repeated twelve times and the duration of each run of the experiment was about 120 seconds. The experiments were repeated on two days to test the reliability and robustness.

### Analysis of sEMG recordings

#### 1. Computing the established features

The first step in the analysis of the data was to compute the following features of sEMG that have been proposed by other researchers. For details, the reader is directed to [9]:

• Root mean Square (RMS)

(1) $$RMS=\sqrt{\frac{1}{N}\sum_{i=1}^{N}x_{i}^{2}}$$

• Mean absolute value (MAV),

(2) $$MAV=\frac{1}{N}\sum_{i=1}^{N}\left|x_{i}\right|$$

• Variance (VAR)

(3) $$VAR=\frac{1}{N}\sum_{i=1}^{N}{\left(x_{i}-\bar{x}\right)}^{2}$$

• Waveform length (WL).

(4) $$WL=\frac{1}{N}\sum_{i=1}^{N-1}\left|x_{i+1}-x_{i}\right|$$

where *N *is the number of samples in a segment and *x*_*i *_is the signal

#### 2. Computing the proposed set of features

The next step was the computation of the proposed set of features; Fractal Dimension (FD) and Maximum Fractal Length (MFL) of sEMG. This is described below:

FD was calculated using Higuchi algorithm [38,39] for non-periodic and irregular time series. This algorithm yields a more accurate and consistent estimation of FD for physiological signals than other algorithms [40].

The first step for computing the MFL requires the computation of the length of the curve, $X_{k}^{m}$, for a time signal sampled at a fixed sampling rate, *x(n) = X(1), X(2), X(3), ....., X(N) *as follows:

(5) $$L_{m}(k)=\frac{\left\{\left(\sum_{i=1}^{\left[\frac{N-m}{k}\right]}\left|X\left(m+ik\right)-X\left(m+\left(i-1\right).k\right)\right|\right)\frac{N-1}{\left[\frac{N-m}{k}\right].k}\right\}}{k}$$

where *[ ] *denotes the Gauss' notation and both *k *and *m *are integers. *m = *initial time; *k*= time interval; i = 1 to $\left[\frac{N-m}{k}\right]$

**Figure 3:**
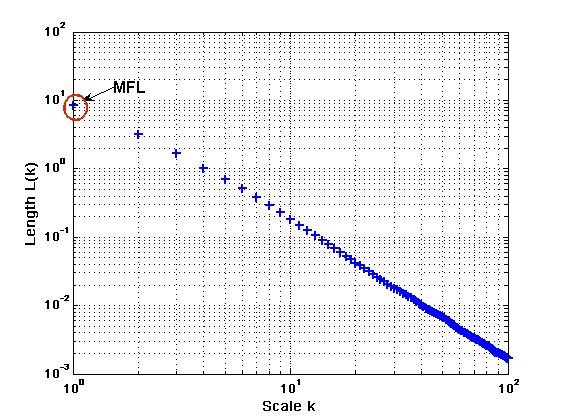
Calculation of Maximum Fractal Length (MFL) and Fractal dimension (FD-slope of the line) from the logarithmic plot of length L(*k*) vs scale *k*

The term $\frac{N-1}{\left[\frac{N-m}{k}\right].k}$ represents the normalization factor for the curve length of subset time series. The length of the curve for the time interval *k*, ⟨*L*(*k*)⟩ is defined as the average value over *k *sets of *L*_*m *_(*k*). If ⟨*L*(*k*)∝*k*^-*D*^⟩, then the curve is fractal with the dimension *D*. Maximum fractal length (MFL) was determined from the plot (Figure 3) as the average length L(*k*) at the smallest scale. The slope of the line gives the fractal dimension (FD). The computation MFL and FD is shown in Figure 3. A threshold, T, was obtained based on the maximum MFL value (after removing the outliers) when there was no hand action. The MFL was compared with T and this was used to determine the onset of muscle activity.

### Feature extraction and classification

A sliding window of 1024 samples corresponding to one second was used for computing the features of sEMG recorded from each flexion. This corresponds approximately to one hertz, which is faster than the normal speed of human finger actions. While this paper reports 1024 sample window, experiments were also conducted where the window size was 512 samples and the outcome was identical. SEMG corresponding to the isometric contraction was analyzed and sEMG corresponding to the action was removed by removing the first and the last one-second of the contraction.

After removing the first and the last second data of each flexion, 5 seconds long recordings corresponding to each flexion were selected. With a window size of 1024 samples, this provided 5 segments for each flexion. The data of all 5 segments and for all the 12 repetitions for each flexion (total number of flexions = 4) was considered for statistical analysis. The data of the 5 subjects were analyzed together. Hence the total sample size of (12*5*5*4 =) 1200 was used for the statistical analysis. It was observed that inter-experimental variations were very significant for RMS compared with other features. Hence the RMS was normalized by taking a ratio of all the channels with respect to channel 1. This resulted in channel 1 becoming redundant and effectively reduced the four channels to three. While computing MFL, the threshold, T was considered for data from every subject individually. The threshold, T did not vary much with the subjects as it was computed from sEMG when the participant performed no hand movement (when the muscle is at rest).

Multi-variant analysis of variance (MANOVA) was conducted to determine the significance of the relationships and to obtain the two out of four most representative channels for each of the features. MANOVA identifies a linear combination of the variables, called the canonical variable, that has the highest multiple correlations with the groups, and these canonical variables provide the order of level of correlation. Statistical analysis was performed to determine the significance of separation of the different features to identify the different actions. A combination of various variables and multiple channels were taken:

• Two channels (selected using MANOVA) for each of the 6 features.

• Four channels for each of the 6 features.

• Single channel with FD paired with each of the other 5 features.

The next step was the classification of each of the feature set listed above using Artificial Neural Network (ANN). The ANN with two hidden layers and 20 neurons for each hidden layer was simulated. The Sigmoid threshold function with a learning rate of 0.05 was used to reduce the likelihood of local minima. The ANN analysis was repeated for each feature sets, with input to the ANN being the sEMG features and the target being the associated actions. ANN was trained using 50% of the data and tested with the other 50% which has not been used for training. Ten cross validation was performed by changing the training and testing data. Classification accuracy was computed as the average accuracy based on the results from cross validation testing. The data of the proposed set of features, FD and MFL of a single channel (Channel 2 is the channel closest to the elbow and represents the condition suitable for a trans-radial amputee) was plotted for visualization to assess the separation of the different classes. This provides a qualitative analysis of the data.

## Results

**Figure 4:**
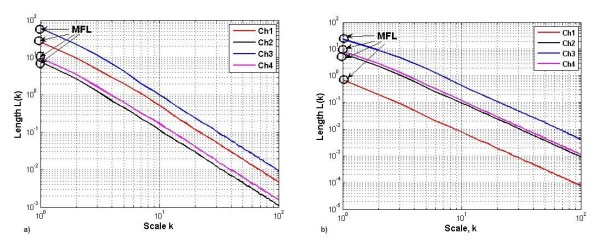
Example representation of MFL for the four channel recorded sEMG signal during two different Wrist flexions a) *M1 *b) *M3*

**Figure 5:**
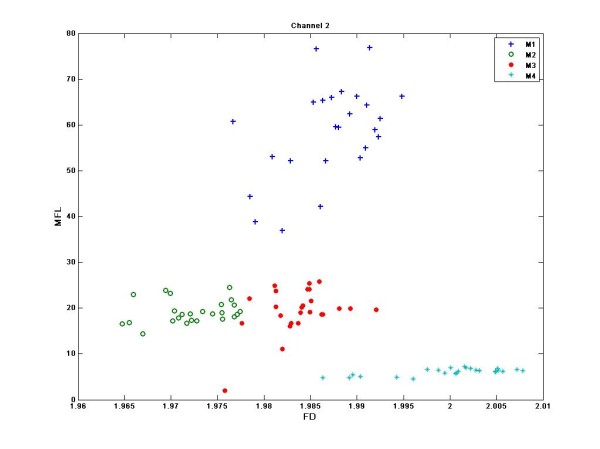
**Grouped Scatter plot of FD and MFL of single channel sEMG**. Channel 2 is shown in this plot.

Figure 4 is the sample representation of the MFL of multiple channels for different wrist flexions. From this figure, it can be observed that the pattern of the MFL is different for the two wrist flexions (M1 and M3). Figure 5 is the two-dimensional plot of the MFL and FD of single channel (channel 2) of sEMG. While all of the channels had similar results, channel 2 was selected for this figure because of its proximity to the elbow making it most suitable for the prosthetic control. It is also observed from figure 4 that each of the actions form distinct clusters indicating clear separation between the different actions.

### Statistical Analysis

From table 1 and table 2, it is observed that for the multiple (two and four) channels, the MFL is more significant than that of the other five features. This clearly demonstrates that MFL of multiple channels is the more reliable feature for identifying the hand gestures. The value of *p *inversely indicates the significance of separation of the classes.

**Table 1:** F-statistic table for two Channels combined (Channel 2 and Channel 3)

F-value	RMS	MAV	WL	VAR	FD	MFL
Average	19.95	41.56	44.50	20.22	34.52	102.21
SD	2.593	4.259	12.738	1.3076	5.259435	5.225
Significance, Average p*	**0.0167**	**0.01**	**0.0099**	**0.017**	**0.01**	**0.004**

* Significance based on 3 degrees of freedom

**Table 2:** F-statistic table for all four Channels combined for different features

F-value	RMS	MAV	WL	VAR	FD	MFL
Average	50.56	80.25	110.26	40.27	65.32	190.58
SD	16.583	19.236	21.246	5.367	10.289	15.259
Significance, Average p*	**0.01**	**0.0095**	**0.008**	**0.01**	**0.009**	**0.001**

*Significance based on 3 degrees of freedom

The results of statistical analysis of single channel sEMG for FD paired with each of the other features are tabulated in table 3. From the results, it is observed that while the value of *p *for FD and MFL combination is 0.0001, the value of *p *for other combinations ranges between 0.02 and 0.199. The *F *value results also indicate that the most suitable feature set is FD and MFL of a single channel. This demonstrates that FD and MFL of a single channel are suitable for identifying the four different hand gestures.

**Table 3:** F-statistic table for single Channel (Channel 2) for various feature sets

F-value	FD & MFL	FD & RMS	FD & MAV	FD & WL	FD & VAR
Average	210.936	93.334	101.33	123.064	50.43
SD	15.778	21.652	27.061	26.211	38.238
Significance, Average p*	**0.0001**	**0.1**	**0.098**	**0.02**	**0.199**

*Significance based on 3 degrees of freedom

### Classification accuracy

The average classification accuracy of multiple (two and four) channels of the six features for identification the associated actions are shown in table 4 and table 5. The average accuracy of each feature paired with FD with only single channel sEMG has been tabulated in table 6. These results reconfirm the above observation based on statistical analysis of the data. MFL and FD of single channel were accurately able to identify the actions with 90.7% accuracy, while the accuracy based on other features (single channel, paired with FD) ranged from 58% to 73%. The comparable accuracy was obtained when using 4 channels MFL.

**Table 4:** Classification Accuracy of the various features using two channels (Channel 2 and Channel 3) sEMG

	RMS	MAV	WL	VAR	FD	MFL
Average	75%	79.33%	81.67%	61.33%	65.33%	83.67%
SD	11.17	10.04	9.55	14.29	8.34	10.26

**Table 5:** Classification Accuracy of the various features using all four channels sEMG

	RMS	MAV	WL	VAR	FD	MFL
Average	80.23%	82.25%	89.33%	68.58%	69.67%	90.33%
SD	10.41	9.23	8.51	12.24	9.57	5.35

**Table 6:** Classification Accuracy of the various features using single channel (Channel 2) sEMG

	FD & MFL	FD & RMS	FD & MAV	FD & WL	FD & VAR
Average	90.67%	68.33%	69.67%	73.35%	58.68%
SD	2.04	7.45	8.26	8.78	5.786

The accuracy using a combination of FD and MFL obtained from single channel was even better than when using all four channels, where the accuracy of identification of the actions ranged between 70% and 90%. This indicates that FD and MFL combination of single channel sEMG was significantly more accurate in identifying finger and wrist flexions compared with any other feature that was tested.

## Discussion and Conclusion

SEMG is a measure of the muscle activity that has been used by many researchers to identify control commands for controlling prosthetic hands and for human machine interface. One shortcoming in the use of sEMG for identifying control commands is the unreliability when the muscle activity is weak and there are multiple active muscles. This is because of the presence of background noise, other artefacts and cross-talk. This study has overcome the above limitations and developed a technique that can reliably identify control commands even when the strength of sEMG is weak, and there are multiple active muscles such as during finger and wrist flexions.

This study has demonstrated that the combined use of FD and MFL of single channel sEMG recorded from the forearm is the most accurate feature set to identify finger and wrist flexion movements when compared with the established features reported in literature. While the features set (FD and MFL) accurately identified finger and wrist flexion movements with average accuracy of 90.7% by comparison the accuracy of identification using other features of the signal reported in literature [9,41] ranged from 61% to 83.7%. The statistical analysis also confirmed the significance of the relationship of FD and MFL with the hand gestures, and the lower significance for all the other features. There was no observable difference of the outcomes for experiments conducted on two different days, indicating that there was insignificant impact of inter-experimental variations on the efficacy of this technique. Small variations that would have been in the location of electrodes between the experiments do not appear to have an impact on the ability of the system to accurately identify the different actions.

Based on the experimental outcomes of this study, it is concluded that a combined use of FD and MFL of single channel sEMG is suitable for reliably identifying various finger and wrist flexion actions without being sensitive to inter-experimental variations and does not require strict electrode positioning. While comparable accuracies are obtainable using number of channels, a single channel is desirable because of lower complexity and it being suitable for amputees who may not have a large area of the forearm available for multi-channel sEMG recording. Such a system can be used for controlling the individual fingers of a prosthetic hand for amputees. The system may be suitable for other applications such as human computer interface for the elderly and for people in special circumstances such as defence.

## Competing interests

The authors declare that they have no competing interests.

## Authors' contributions

SPA has conducted the experiments, developed the signal processing technique and performed the data analysis. He has also written the first draft of the manuscript. DKK has designed the experiment, and discussed and developed the underlying concepts for the technique. He has also done the proof-reading, and finalized the manuscript. All authors have read and approved the manuscript.
